# Diet-Induced Obesity Reduces the Responsiveness of the Peripheral Taste Receptor Cells

**DOI:** 10.1371/journal.pone.0079403

**Published:** 2013-11-13

**Authors:** Amanda B. Maliphol, Deborah J. Garth, Kathryn F. Medler

**Affiliations:** Department of Biological Sciences, University at Buffalo, The State University of New York, Buffalo, New York, United States of America; University of Tokyo, Japan

## Abstract

**Introduction:**

Obesity is a growing epidemic that causes many serious health related complications. While the causes of obesity are complex, there is conclusive evidence that overconsumption coupled with a sedentary lifestyle is the primary cause of this medical condition. Dietary consumption is controlled by appetite which is in turn regulated by multiple neuronal systems, including the taste system. However, the relationship between taste and obesity has not been well defined. Growing evidence suggests that taste perception in the brain is altered in obese animals and humans, however no studies have determined if there are altered taste responses in the peripheral taste receptor cells, which is the initiation site for the detection and perception of taste stimuli.

**Methodology/Principal Findings:**

In this study, we used C57Bl/6 mice which readily become obese when placed on a high fat diet. After ten weeks on the high fat diet, we used calcium imaging to measure how taste-evoked calcium signals were affected in the obese mice. We found that significantly fewer taste receptor cells were responsive to some appetitive taste stimuli while the numbers of taste cells that were sensitive to aversive taste stimuli did not change. Properties of the taste-evoked calcium signals were also significantly altered in the obese mice. Behavioral analyses found that mice on the high fat diet had reduced ability to detect some taste stimuli compared to their littermate controls.

**Conclusions/Significance:**

Our findings demonstrate that diet-induced obesity significantly influences peripheral taste receptor cell signals which likely leads to changes in the central taste system and may cause altered taste perception.

## Introduction

While obesity is a complex disease, it is fundamentally due to overconsumption [1], [2]. Our sense of taste can impact consumption since it functions to identify potential food items [3], [4]. Food consumption is strongly influenced by appetite and the hormones that regulate appetite can also affect taste [5]–[7]. Thus, the taste system is a potentially important target in appetite regulation. Taste perceptions in obese humans and rodents can be altered which likely affects their consumption [8]–[10]. Previous studies have focused on the central taste system but to date, no studies have determined if the signals in the peripheral taste receptor cells are altered in obese organisms. Understanding the role of the peripheral taste system in obesity is important because detecting potential nutrients is the first step in food consumption.

Taste determines whether prospective food items will be ingested or rejected. Sweet and umami tastes are appetitive and are used to detect nutrient rich foods. Bitter taste is aversive and identifies potentially harmful compounds to avoid [11]. Multiple studies have reported that the ability to detect these taste stimuli is altered in obese humans and rodents [8], [12]–[16]. While most studies have focused on changes in the sweet preferences due to obesity, a few studies have focused on umami and bitter perception in obese as well [10], [17]. Research on sweet and umami preference in humans revealed that obese individuals may not taste these stimuli as well, but have increased preferences for them compared to their lean counterparts [8], [17]. One possible explanation is that these individuals cannot detect appetitive compounds as well and require more to reach satiety. However, so little is known about the cellular mechanisms that contribute to obesity that this hypothesis has not been directly tested. Other studies have shown no differences in sweet preferences between obese and lean individuals [18], [19]. Similarly conflicting findings also exist from studies examining the relationship between bitter perception and body mass [20], [21]. This is likely because both taste perception and obesity are complex processes that are regulated by multiple systems.

If taste signals are altered, feeding behaviors could be significantly impacted which could contribute to the development of obesity. Using calcium imaging to measure taste-evoked signals in peripheral taste receptor cells of obese mice, we found that multiple taste signals are significantly reduced compared to control littermates, suggesting that the responsiveness of the peripheral taste receptor cells is suppressed in obese mice. Behavioral analysis also reveals that obesity can affect some taste preferences. Since nothing is currently known about the relationship between the peripheral taste receptor cells and obesity, this study is the first to demonstrate that diet-induced obesity significantly alters the responsiveness of the peripheral taste cells that are responsible for the initial detection of taste stimuli and for sending that taste information to the brain.

## Materials and Methods

### Weight Monitoring

All animal studies were approved by the University at Buffalo Animal Care and Use Committee under protocol number #BIO010174N. C57BL/6 mice were taken from litters that were born within a week of each other. One month after weaning, half of the mice (n = 25) from each litter were placed on high fat mouse chow (60% high fat Kcal feed, Harlan Labs, Inc., Madison, WI, USA; diet is comprised of 60% calories from fat, 22% calories from carbohydrates, 18% calories from protein) while the remaining littermates (n = 25) were kept on normal mouse chow (Harlan labs: diet is comprised of 18% calories from fat, 58% calories from carbohydrates, 24% calories from protein). Initial weights of the mice were taken and mice on the high fat diet were measured once a week for 16 weeks. Weekly measurements of the mice on the normal chow began at week 2. Average values with the standard error of the mean are reported.

### Behavioral Procedures

To determine if diet-induced obesity affects the taste preferences of the C57BL/6 mice, mice from each group were subjected to two bottle preference tests using the protocol described [22]. All solutions were prepared daily with distilled water and were presented at room temperature. Each test concentration was presented in conjunction with water for a total of 48 h. Test solutions were switched with water every 24 h to ensure no side preferences developed. Preference ratios were calculated as volume of test solution intake/total volume intake (test solution+water). Five taste qualities were tested with each test condition having 5 mice on the fat diet and 5 control mice. All behavioral assays were performed on naïve mice and two bottle preference tests were run concurrently to minimize any potential bias due to time differences. Thus, all behavioral experiments were completed within two weeks.

Five taste stimuli were tested: acesulfame K (AceK), sucrose, saccharin, monopotassium glutamate (MPG), and denatonium (Den). Each stimulus was presented at four concentrations in ascending order. The concentrations used were as follows (in mM): 1) AceK 1, 2, 20, 50; 2) sucrose 5, 50, 150, 300; 3) saccharin 1, 2, 10, 20; 4) MPG 10, 30, 100, 300; 5) Den 0.1, 0.5, 1, 10. Preference ratios for obese and control mice were compared using repeated measures two-way ANOVA with a Bonferroni’s *post-hoc* analysis and Student’s *t*-tests were used to determine significant differences for each concentration. Average values are plotted with standard deviation for each stimulus concentration. A value of p<0.05 was determined as the limit of significance.

### Calcium Imaging

All measurements of intracellular calcium were performed in isolated taste cells. Taste receptor cells were harvested from the circumvallate and foliate papillae from adult C57BL/6 mice and were isolated from lingual epithelium as previously described [22], [23]. All chemicals were purchased from Sigma Chemical (St. Louis, MO, USA) unless otherwise noted. Isolated taste receptor cells were plated into a laminar flow chamber and loaded at room temperature for 40 min with 2 µM fura 2-AM (Molecular Probes, Invitrogen) containing the nonionic dispersing agent Pluronic F-127 (Molecular Probes, Invitrogen). Loaded cells were visualized using an Olympus IX71 microscope with a 40× oil-immersion lens and images were captured using a SensiCam QE camera (Cooke, Romulus, MI, USA) and Workbench 5.2 (Indec Biosystems, Santa Clara, CA, USA). Excitation wavelengths of 340 and 380 nm were used with an emission wavelength of 510 nm.

### Data Analysis

Experimental results were plotted and analyzed using OriginPro software (OriginLab Corp., Northampton, MA, USA). An evoked response was defined as measurable if the increase in fluorescence was more than two standard deviations above baseline. Calcium increases were calculated as [(peak − baseline)/baseline]×100 and were reported as percentage increases over baseline to determine the response amplitudes. We integrated the area under the curve to obtain a proportional measure of the amount of calcium in a response. The entire area under the curve was analyzed beginning with the initial change in baseline calcium and measuring until the levels returned to comparable baseline values. If the response did not return to baseline calcium levels, it was not included in the analysis. Only evoked responses were included in the analyses, null responses were not included. Statistical comparisons were made using either Student’s *t*-test or one-way ANOVAs with a Bonferroni’s *post-hoc* analysis. Frequency values were analyzed using a Chi-square analysis [24] to determine if there were any significant differences in the responsiveness of taste cells isolated from obese mice compared to control mice. For all calcium imaging analyses, a significance level of p<0.05 was used and standard errors of the mean were reported.

### Solutions

All solutions used for calcium imaging were bath applied using a gravity flow perfusion system (Automate Scientific, San Francisco, CA, USA) and laminar flow perfusion chambers (RC-25F; Warner Scientific, Hamden, CT, USA). The following tastants were diluted into Tyrode’s and were used to stimulate the cells during experiments: 2 mM saccharin, 5 mM denatonium benzoate, 20 mM acesulfame K, 20 mM monopotassium glutamate.

## Results

### Taste Receptor Cells in Obese Mice are Less Responsive to Appetitive Taste Stimuli

**Figure 1:**
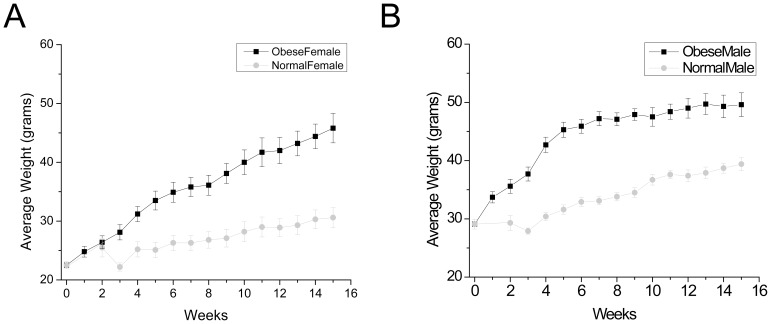
Mice on a high fat diet become obese compared to control mice. After two weeks, mice on the high fat diet (black line) began to show rapid weight gain compared to their littermate controls on normal diet (gray lines), n = 25 for each group. Weights were recorded weekly and the average weights are reported here with standard error bars (SEM) for both female (A) and male (B). By week two, males on the high fat diet are significantly heavier than their male littermate controls (p<0.01) and continued to be significantly heavier throughout the rest of the study. By week three, female mice on the high fat diet were significantly heavier than their female littermate controls (p<0.01) and remained significantly heavier for the rest of the study.

C57Bl/6 mice (n = 25) were placed on a high fat (60% high fat kcal feed) diet within one month of weaning while littermate controls (n = 25) were kept on normal mouse chow. Weights were measured and recorded weekly for all mice and are shown in Figure 1. Mice on the high fat diet rapidly gained weight compared to the control mice on normal mouse chow. After eight weeks on the high fat food, mice were behaviorally analyzed using two bottle preference tests to determine if there were any effects of diet and/or weight gain on the preference ratios. All experiments were performed on naïve mice (n = 5 obese and n = 5 control mice for each taste quality) and then these mice were used for the subsequent calcium imaging experiments. Calcium imaging experiments were evenly switched between obese mice and control mice to reduce any potential bias due to differences in timing of the imaging experiments.

After 10 weeks on the high fat diet, mice were between 30% (males) and 40% (females) heavier than controls. Beginning at week 10, we isolated taste receptor cells and analyzed the taste-evoked calcium signals from both obese and control mice. We tested three appetitive taste stimuli, including two sweet stimuli (2 mM saccharin-SAC and 20 mM Acesulfame K-Ace K) and one umami stimulus (20 mM monopotassium glutamate-MPG). We did not test any sugars since their effective concentrations could potentially have non-specific osmotic effects on the isolated taste cells. While the artificial sweeteners we used have been reported to generate a bitter aftertaste at higher concentrations [25]–[27], our behavioral assays demonstrate that the mice used in this study do not avoid these sweeteners at the concentrations used in the calcium imaging experiments. Thus, we considered these evoked calcium responses as sweet responses. We also measured responses to an aversive bitter compound (5 mM denatonium-Den). We used these concentrations for each of the taste stimuli based on earlier studies in which we determined the optimal concentration needed to evoke a maximal calcium signal for each stimulus in normal C57Bl/6 mice (unpublished data). Since we were comparing evoked calcium signals to taste stimuli from both groups, it was important to compare responses at maximal effective concentrations to ensure that we did not confound our data with sub-maximal evoked responses which could be more variable.

**Figure 2:**
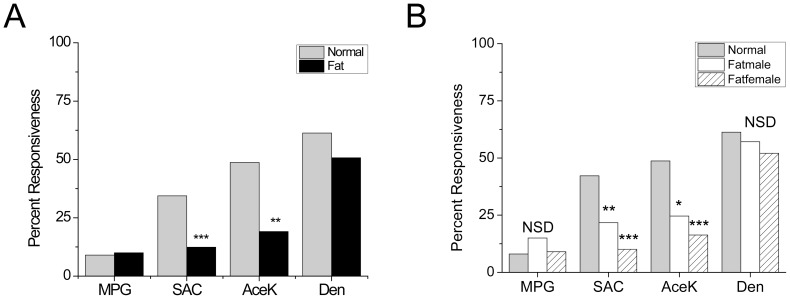
Fewer taste cells are sensitive to sweet taste stimuli in obese mice. Chi-square analysis was used to compare the percentage of responsive taste receptor cells between normal and obese mice. (A) Bar graphs represent the percentages of taste-evoked calcium responses to each taste stimuli. The number of responsive taste cells for the sweet stimuli (2 mM Sac, 20 mM AceK) were significantly reduced for the obese mice (black bar) compared to controls (gray bar) (***, p<0.001; **, p<0.01; *, p<0.05). The numbers of taste-evoked calcium responses to the umami taste stimuli (20 mM MPG) and the aversive bitter stimuli (5 mM Den) were not significantly different between the two groups. (B) When obese mice were analyzed by sex, both males and females were significantly less responsive to the sweet taste stimuli tested compared to control mice (***, p<0.001; **, p<0.01; *, p<0.05).

For each taste stimulus, we generated response profiles for the obese and control mice. The goal of these experiments was to determine if there were any differences in the responsiveness of the taste receptor cells to the taste stimuli as well as to detect any differences in the taste-evoked responses between the obese and normal mice. Thus we did not determine which taste cell type was responding to the taste stimuli, we only measured the taste-evoked calcium signals in each responsive taste cell. The exception to this was the umami (20 mM MPG) responses. Since MPG could potentially activate any neurotransmitter glutamate receptors expressed in the Type III cells [28], [29] which form conventional synapses and express voltage-gated calcium channels [30], [31], we restricted our analysis to MPG evoked responses that were found in the taste cells that did not respond to 50 mM KCl. Taste cells that were sensitive to 50 mM KCl were presumed to express voltage-gated calcium channels (VGCCs) and be Type III taste cells. Since MPG contains potassium, it is also possible that application of 20 mM MPG could depolarize the cell enough to activate VGCCs and generate a calcium response. Thus taste cells that responded to 20 mM MPG but did not express VGCCs were designated as Type II taste cells and the calcium responses from these cells were considered umami responses. The overall percentages of responsive cells for each taste stimulus in the obese and normal mice are shown in Figure 2A. We used a Chi-Square analysis [24] to determine if the response frequencies were significantly different between the obese mice and their littermate controls. The percentages of responsive taste cells for the sweet taste stimuli were significantly higher in the control mice compared to the obese mice. We found no significant differences for the response profiles to the umami or bitter stimuli. When the data from the obese mice was separated into male and female components, we found that both male and female mice had significantly reduced response profiles for the sweet stimuli compared to controls (Figure 2B). Individual comparisons between the obese males and females for the taste stimuli revealed that obese females had significantly fewer saccharin responsive (p<0.01) taste cells compared to obese males. There were no significant differences between the sexes for the other taste stimuli (Figure 2B). These data indicate that the peripheral taste cells are less responsive to sweet taste stimuli in obese mice compared to control mice and that these effects can be more severe in the obese female mice compared to the obese males.

**Figure 3:**
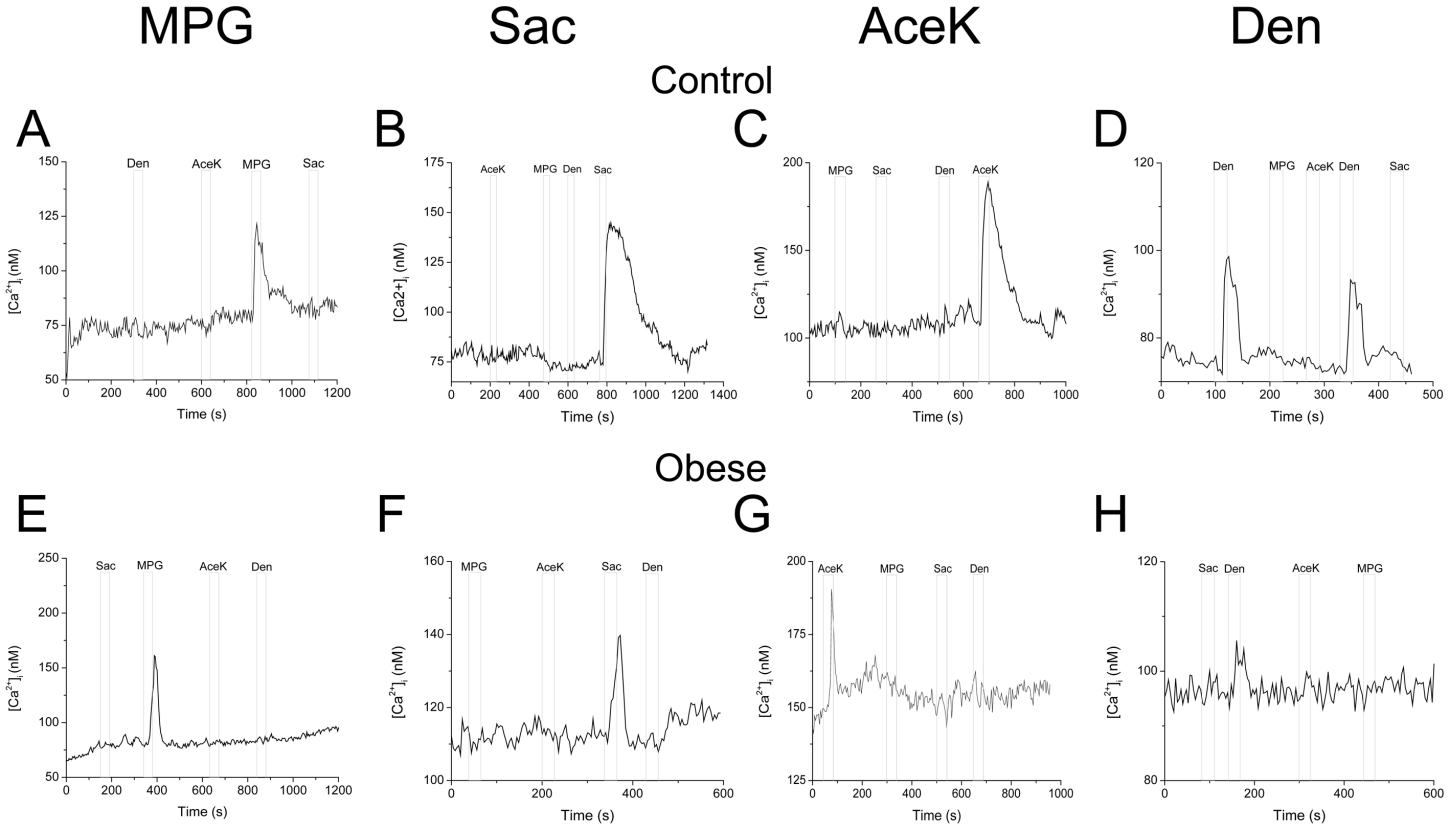
Taste evoked calcium signals are altered in obese mice. Representative taste evoked calcium signals are shown from both control (A–D) and obese (E–H) mice for multiple taste stimuli: umami (20 mM MPG, A, E); sweet (2 mM Saccharin, B, F and 20 mM AceK, C, G); and bitter (5 mM denatonium, D, H).

**Figure 4:**
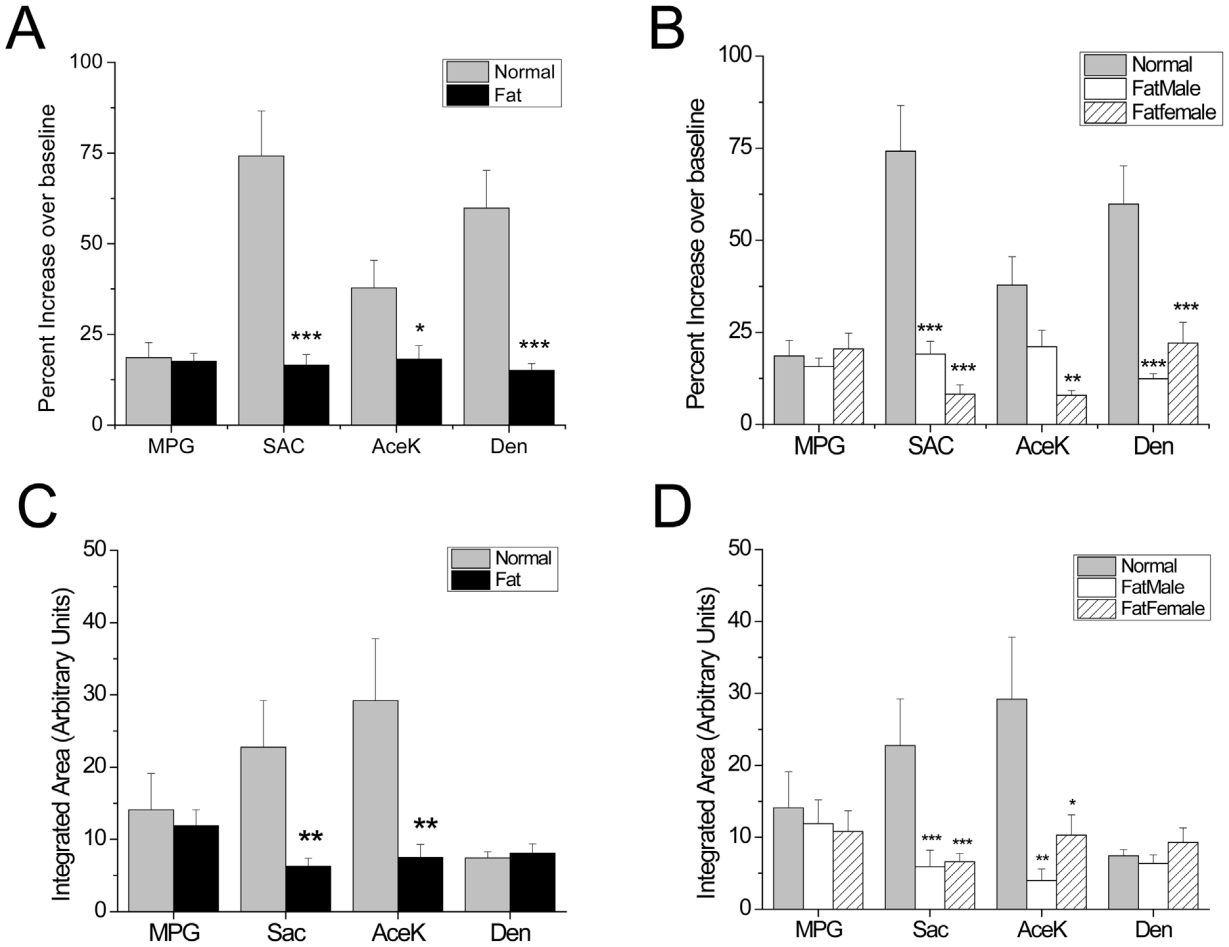
Calcium signals are altered in obese mice. (A) Amplitudes of the taste-evoked calcium responses were measured as a percent increase over baseline. Amplitudes were significantly reduced in obese mice for the sweet stimuli tested (2 mM Sac, 20 mM AceK) and one aversive stimulus, denatonium (5 mM). Response amplitudes of the measured umami (20 mM MPG) stimuli were not different between obese mice and their littermate controls. (B) Response amplitudes were significantly lower in both obese males and obese females for the sweet and bitter stimuli tested (***, p<0.001; **, p<0.01) with the exception of the AceK responses in obese males (p = 0.06). Average peak values (% increase over baseline) for each stimulus as shown in B: MPG, Ctl = 18.6, males = 15.7, females = 17; SAC, Ctl = 74.2, males = 19.1, females = 8.2; ACEK, Ctl = 37.8, males = 21.1, females = 7.9; DEN, Ctl = 59.8, males = 12.4, females = 22.1. (C) A proportional measurement of calcium signals were represented by the integrated area under the curve. The overall calcium responses to the sweet stimuli were significantly smaller in obese mice compared to controls. There was no difference found for the umami stimulus (20 mM MPG) or the bitter stimulus (5 mM denatonium). (D) The integrated areas of taste evoked responses for the obese males and obese females were significantly reduced for the sweet taste stimuli tested compared to control responses (***, p<0.001; **, p<0.01; *, p<0.05). No differences were found for the umami stimulus, MPG or the bitter stimulus, denatonium. Average AUC values (arbitrary units) for each stimulus as shown in D: MPG, Ctl = 14.1, males = 11.9, females = 10.8; SAC, Ctl = 22.8, males = 5.9, females = 6.6; ACEK, Ctl = 29.2, males = 4, females = 10.3; DEN, Ctl = 7.4, males = 6.4, females = 9.3.

Not only were the numbers of responsive taste cells reduced, but the properties of the calcium signals were also significantly altered in the obese mice compared to controls. Representative traces of the different taste-evoked calcium responses are shown in Figure 3 for both control mice (A–D) and the diet-induced obese mice (E–H). Analysis of the taste-evoked calcium responses revealed some significant differences between the obese mice and their littermate controls. The amplitudes of the responses were significantly reduced in the obese mice for the sweet taste stimuli tested (Figure 4A). Surprisingly, the amplitudes of the denatonium responses from the obese mice were also significantly reduced compared to controls (n = 21 for Ctl, n = 29 for obese males, n = 13 for obese females). No differences were seen in the umami responses (n = 24 for Ctl, n = 26 for obese males, n = 17 for obese females). Separating out the data collected from the male and female mice, we found that almost all of the response amplitudes were comparably reduced in both male and female obese mice for the sweet and bitter stimuli (Figure 4B, SAC, n = 18 for Ctl, n = 8 for obese males, n = 16 for obese females). The only exception was the amplitude of the obese males to the sweetener acesulfame K which was not significantly different from control (n = 14 for Ctl, n = 18 for obese males, n = 10 for obese females, p = 0.06). Values for each taste stimulus were compared using one-way ANOVA with a Bonferroni’s post-hoc analysis.

We also integrated the area under the curve to get a proportional measurement of the overall calcium signals using the same cells that were analyzed in Fig. 4B and found that for sweet stimuli analyzed, obese mice had significantly smaller taste-evoked calcium responses compared to controls. The bitter-evoked calcium signals were not different from each other (Figure 4C). When we separated the obese male and female responses, there were no significant differences for the integrated areas of the taste-evoked responses between them (Figure 4D). Values for each taste stimulus were compared using one-way ANOVA with a Bonferroni’s post-hoc analysis. In an effort to determine how the peak responses of the dentatonium evoked calcium signals could be significantly reduced in the obese mice while the overall proportional signal was not different, we also analyzed the response duration for these signals. We found that the denatonium-evoked signals were significantly longer in the obese mice compared to controls (obese: average duration = 65.5 s; control: average duration = 50.5 s, p<0.002).

### Weight Gain Affects the Ability to Detect some Taste Stimuli

After 8 weeks, mice on the high fat food were about 35% heavier than their littermate controls (Figure 1). At this time, we performed behavioral assays to determine if there were any differences in the ability of the obese mice to detect taste stimuli compared to the control mice. For these experiments, we tested the same tastants that were used in the calcium imaging experiments. We also added another sweet stimulus, sucrose. For each taste stimulus tested, we used five mice on the high fat diet and five mice on normal chow.

**Figure 5:**
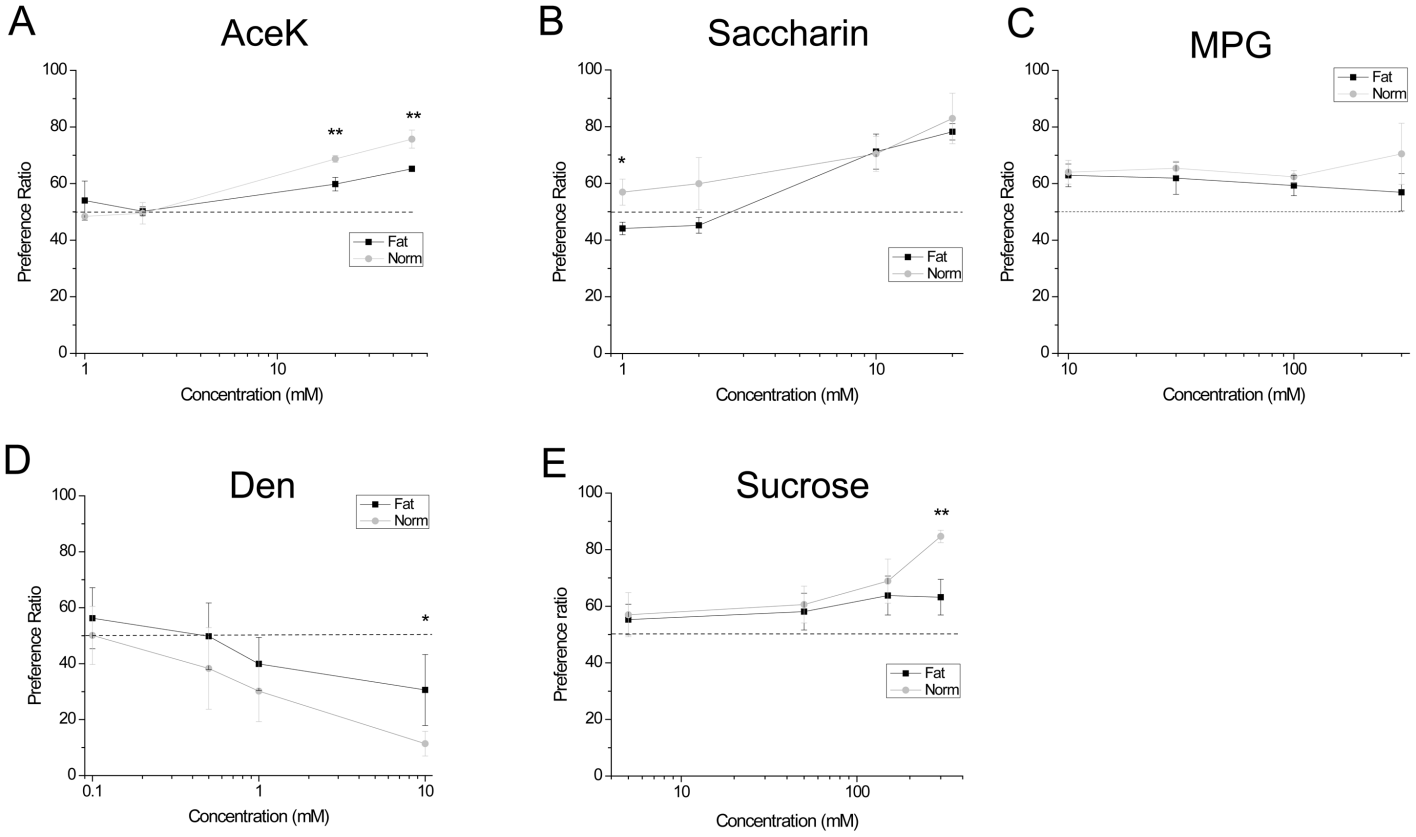
Preference ratios for taste stimuli are altered in obese mice. (A) For the sweet stimulus AceK, the ability to detect this sweetener at higher concentrations was impaired in obese mice compared to controls (**, p<0.01). (B) For saccharin, obese mice were significantly impaired in their ability to detect this sweet stimulus at the lower concentrations tested (*, p<0.05) but were not significantly different in their preferences from controls at higher saccharin concentrations. (C) Control mice showed an increased preference for the umami stimulus, MPG, at the highest concentration but no significant differences between normal and obese mice were measured (p = 0.056). (D) The ability to avoid denatonium at the highest concentration was compromised in obese mice (*, p<0.05) compared to littermate controls. (E) Obese mice were comparable to wild type control mice in their preference for sucrose except for the highest concentration of sucrose tested (300 mM). At this concentration, control mice had a very strong significantly higher preference for sucrose compared to the obese mice.

Since we used two bottle preference tests, there is potential for post-ingestive effects in these experiments. In particular, the nutritive sweet stimulus, sucrose is noted for having post-ingestive effects. For some experiments, the short-exposure lickometer test is preferred to avoid any potential post-ingestive effects [32]. However, recent studies have shown that nutrient rich solutions can generate post-ingestive effects very early after testing begins, within 5 minutes of the testing onset [33]. Thus, even lickometer testing can be influenced by post-ingestive effects which could potentially confound the results. Consequently, we chose to use the longer testing period to avoid any potential training issues associated with the brief-access lickometer experiments and we tested multiple types of compounds that would be unlikely to have the same type of post-ingestive effects [34]. The obese mice were generally less sensitive to the tested taste stimuli compared to mice kept on normal chow. For some sweet stimuli (sucrose, AceK), obese mice were significantly reduced in their preference for the higher concentrations of the stimuli (Figure 5A, E) but were not different from control at lower concentrations. However, for saccharin (Figure 5B), obese mice were less sensitive than controls at low concentrations but were comparable in their preferences at higher concentrations. For the umami stimulus tested (MPG, Figure 5C), no significant differences between obese and control mice were detected, though the control mice did show a preference for MPG at the highest concentration tested that was not seen in obese mice. However, these values were not significantly different. Obese mice did not avoid denatonium as well as control mice at the highest concentration tested (Figure 5D).

## Discussion

### A High Fat Diet Significantly Changes the Rate of Weight Gain in C57Bl/6 Mice

Our data clearly show that a high fat diet has a significant impact on the peripheral taste system, either directly or as a consequence of the subsequent weight gain. We placed age-matched C57Bl/6 mice on either a high fat diet or regular mouse chow. Regardless of the diet, all mice gained weight during the experiment since they were relatively young (7–8 weeks old) when the experiment began. However, the mice on the high fat diet rapidly gained weight and became obese (Figure 1). Male mice also gained weight at a faster rate than the females. After two weeks on the high fat diet, male mice were significantly heavier than the control males (p = 0.0017) while the female mice on the high fat diet were not different from female controls (p = 0.645). However by week three, females on the high fat diet were significantly heavier than the controls (p = 0.004) and remained significantly heavier throughout the study (Figure 1).

### Peripheral Taste Signals are Significantly Reduced in Obese Mice

Many studies have established that processing of taste information in the central nervous system is altered as obesity develops [3], [9], [13], [15], [35]. Growing evidence suggests that hormones which regulate appetite and food intake, including glucagon-like peptide (GLP-1), leptin, endocannabinoids, and vasoactive intestinal peptide (VIP), also affect taste sensitivities. These studies focused on sweet taste using knock out animals, behavioral assays, and cranial nerve recordings to evaluate the hormonal effects on taste responses [7], [36]–[43] and support the hypothesis that the taste system is significantly influenced by hormones associated with appetite. However, none of these studies focused on the initial taste-evoked signals in the taste receptor cells. Since this is the first signal generated in response to a taste stimulus, it is critical to understand how these signals are affected by obesity.

After 10 weeks on the high fat diet, we analyzed the taste-evoked calcium signals in isolated taste receptor cells. Our data reveal significant changes in these peripheral taste signals associated with obesity. Isolated taste cells from obese mice were compared to taste cells from controls to determine their overall responsiveness to different types of taste stimuli. We also measured the response amplitude and integrated the area under the curve to get a measure of how the overall taste-evoked calcium signal was impacted. What we found was quite striking. The number of taste cells that were sensitive to the appetitive sweet taste stimuli was significantly reduced in the obese mice but their responsiveness to the aversive taste stimulus was unaffected. Responsiveness to the umami stimulus tested was also unchanged in the obese mice. Obese female mice had significantly fewer responsive cells to the sweet stimuli compared to the obese male mice (Figure 3). Since males were heavier than females, these data indicate sex-related differences in their sensitivity to obesity-related effects which are not directly due to the amount of weight gained. Thus, the reduction in the number of responsive taste cells does not simply correlate with weight, but appears to be the result of a multitude of factors that are affected by obesity.

Not only were fewer taste cells from the obese mice sensitive to the sweet stimuli, but within the responsive cells, the evoked signals were significantly reduced in both peak amplitude and overall area. Thus, the taste receptor cells appear to lose the ability to respond appropriately to these types of stimuli. Similar results were seen in mice fed a high fat diet that were exposed to long chain fatty acids. The overall calcium signal to the lipids was also reduced in the obese mice compared to control [44], further supporting our conclusion that the taste receptor cells in obese mice are no longer able to respond appropriately to certain chemical stimuli. We did not see a reduction in the umami-evoked responses at any of the measures that we took to analyze the sample. There was no difference in the frequency of MPG responsive cells, the amplitude of the responses did not change and neither did the overall calcium signal. When tested with the two bottle preference test, we also did not detect any significant differences in the responses to MPG between the obese and control mice. Therefore, while umami is generally considered to be an appetitive taste, it was not affected by the high fat diet or the resulting obesity. What we did find surprising, was that the amplitude of the bitter (denatonium) response in the obese mice was significantly reduced (Figure 4) while the overall bitter-evoked signal and the frequency of responsiveness to this stimulus was not different from controls. Further analysis of these signals found that the duration of the bitter response was significantly longer in the obese mice compared to control which explains why the peak signals were significantly reduced but the overall magnitude of the calcium signal did not change. Thus the characteristics of the denatonium evoked signals were significantly altered by diet-induced obesity even though the overall calcium signal did not change. These data suggest that some aspect of the signaling pathway that generates the calcium signal (in this case, the PLCβ signaling pathway) is altered in the obese mice. While the exact mechanism of change is likely a multi-faceted process which is beyond the scope of this current study to characterize, we predict that changes in the appetite regulating hormones which have been shown in other studies to affect taste sensitivities [7], [36]–[43] are likely causing the changes in the taste evoked calcium signals that we measured. While we cannot demonstrate a direct link between the measured changes in the evoked calcium signals and the alterations in the behavioral responses that we found, it is interesting to speculate that altering the characteristics of the evoked calcium signals is sufficient to affect the preference ratios, even if, as seen in the denatonium responses, the overall magnitude of the evoked calcium signal has not changed. Future studies are needed to determine how these alterations in the taste cell signaling pathways develop in response to obesity.

Thus, these data clearly illustrate that at least some of the taste-evoked calcium signals in taste receptor cells are significantly affected in obese mice. These findings are remarkable because the only difference between the obese C57Bl/6 mice and their control littermates was the high fat diet which caused significant weight gain. It is also important to note the selectivity of the obesity effect on the peripheral taste cells, at least for the taste stimuli that we tested. In this study, we only analyzed one bitter compound and one umami compound in addition to the two artificial sweeteners that were tested. For these taste stimuli, the overall responsiveness to bitter and umami taste stimuli was not affected, supporting the conclusion that obesity is not generating a complete suppression of the peripheral taste cell sensitivity but is instead having specific effects on the ability of the cells to respond to certain appetitive taste stimuli. Further comprehensive studies that analyze more taste stimuli, in particular more bitter and umami stimuli, may find different effects. Indeed, our data for denatonium suggests that bitter stimuli can be affected by obesity. The effects were not as severe for the bitter stimulus as was seen for the sweet stimuli tested, but further studies are needed. Taken together, our data demonstrate that obese mice have significant reductions in their responsiveness for at least some taste stimuli.

Different taste stimuli activate distinct signaling pathways in taste cells. Regardless of the signaling mechanism, however, an increase in cytosolic calcium is needed to cause neurotransmitter release and is a critical component in the stimulus response from all taste cells. Recent studies have shown that cytosolic calcium changes in taste receptor cells are directly responsible for transducing all five taste qualities and directly affect the subsequent cranial nerve response [45], [46]. Earlier studies have shown that cranial nerve responses from genetically-induced obese mice are significantly impacted in their ability to transmit evoked taste responses [47], [48] but our findings suggest that the initial peripheral calcium response may be the proximal cause for these observed changes. Thus, some of the obesity-induced changes in the central processing of taste stimuli [3], [9], [13], [35] may be due to the changes in the peripheral taste cell properties. Future studies are needed to directly answer this question.

### Preference Ratios are Significantly Affected by Obesity

After the mice had been on the high fat food for 8 weeks, we performed a two bottle preference test to determine if the ability of the mice to detect different tastants was affected by their obesity. Obese mice had less preference for the sweet stimuli tested (AceK, sucrose and saccharin) compared to controls. In another study, diet-induced obese rats were also less sensitive to saccharin at higher concentrations when compared to control rats [49]. In the same study, rats that were on a calorie restricted diet had a greater preference for saccharin than both the obese and control rats. Other sweet stimuli were not tested. Real time PCR analysis of known sweet receptors (T1R2 and T1R3) and signaling components of the Type II cells from these three groups found that only T1R3 was significantly reduced in the rats on the high fat diet [49]. While the reduced T1R3 mRNA levels in the obese rats were only correlative with the reduced saccharin preference in this study and potential changes in protein levels were not measured, it is interesting to speculate that a reduction in this component of the sweet receptor could be causing the reduction in the saccharin preference by the obese rats. Indeed, T1R3-KO mice are insensitive to saccharin in two bottle preference tests [50] which lends further support to that hypothesis. Further studies are needed to determine if T1R3 levels are also reduced in diet-induced obese mice. These findings are in contrast to another study which found that obese rats have a greater preference for sugars compared to their lean counterparts. This study was done in OLETF rats which lack a functional cholescystokinin receptor that affects satiety [51]. In addition, genetically obese mice also showed an increased preference for sweet stimuli and a stronger aversive response to a bitter stimulus compared to controls. These mice lacked a functional bombesin receptor which is normally expressed in the hypothalamus region that regulates taste perception and feeding behavior [52]. Interestingly, gustatory nerve responses in rats that have developed obesity as a result of ventromedial hypothalamus lesions were also enhanced in response to sugars compared to controls [15]. Taken together, these data suggest that diet-induced obesity and genetically induced obesity may have different effects on the functions of the taste system.

In our study, obese mice were generally insensitive to the umami stimulus tested (MPG) but these values were not significantly different from controls. These findings agree with earlier studies that reveal a general indifference to MSG in naïve mice [53]. Interestingly, the obese mice did not avoid high concentrations of bitter (denatonium) as well as control mice. Thus we conclude that diet-induced obesity causes significant changes in the behavioral preferences of mice, primarily but not exclusively, for appetitive stimuli.

While we cannot conclude that these behavioral changes are directly due to the changes in the taste-evoked calcium signals that we measured in the obese mice compared to controls, our findings do reveal a strong correlative relationship between the responsiveness of the peripheral taste receptor cells and the behavioral responses. The behavioral differences we found between the obese and control mice for the different taste stimuli correlate with the concentrations of those tastants that we used in the calcium imaging experiments. While future studies are required to determine if a causative relationship exists, we can conclude that diet-induced obesity causes a significant repression of the peripheral taste system’s ability to respond to sweet stimuli and that behavioral preferences to these sweet stimuli are also significantly reduced. Thus future studies on the effects of obesity on central taste processing should consider that the initial peripheral taste signals are impacted by obesity which are likely affecting those central pathways and may ultimately be affecting taste perception.
